# Enhancing uptake of PrEP for her via organizational research and individual actions (EUPHORIA): Protocol for a stepped-wedge cluster randomized trial

**DOI:** 10.1371/journal.pone.0355623

**Published:** 2026-08-27

**Authors:** Amy K. Johnson, Samantha A. Devlin, Cristina M. A. Barkowski, Amanda Goldstein, Emory Latz, Rebecca Campbell, Jiehuan Sun, Brenda Wolfe, Madeline Quasebarth, Ariyana Safarloo, Jessica P. Ridgway, Sadia Haider

**Affiliations:** 1 The Potocsnak Family Division of Adolescent and Young Adult Medicine, Ann & Robert H. Lurie Children’s Hospital, Chicago, Illinois, United States of America; 2 Section of Infectious Diseases and Global Health, Department of Medicine, University of Chicago, Chicago, Illinois, United States of America; 3 Department of Obstetrics and Gynecology, Rush University Medical Center, Chicago, Illinois, United States of America; 4 Division of Epidemiology and Biostatistics, University of Illinois Chicago School of Public Health, Chicago, Illinois, United States of America; 5 Planned Parenthood of Illinois, Chicago, Illinois, United States of America; PLOS: Public Library of Science, UNITED KINGDOM OF GREAT BRITAIN AND NORTHERN IRELAND

## Abstract

**Background:**

Black/African American cisgender women (hereafter referred to as “Black women”) experience one of the highest incidences of HIV yet are often not prioritized in HIV prevention initiatives. For example, pre-exposure prophylaxis (PrEP) for HIV prevention continues to be under-prescribed to women despite its proven effectiveness and demonstrated impact on mitigating HIV transmission. Increasing PrEP initiation and persistence among women through tailored implementation strategies remains crucial to HIV prevention efforts, yet few studies have been designed for women, particularly within the context of family planning centers. This article outlines the study protocol to evaluate the effectiveness of implementation strategies (“EUPHORIA”) to improve PrEP care continuum outcomes and to address barriers to PrEP uptake among women in family planning centers in Cook County, Illinois.

**Methods:**

This study builds upon previous work to adapt implementation strategies for increasing PrEP uptake among Black women. The intervention will consist of implementing a combination of strategies and measuring the impact on PrEP utilization among Black women at 8 family planning centers via a stepped-wedge cluster randomized trial. A mixed-methods approach will be utilized to evaluate implementation outcomes and assess the feasibility, acceptability, and effectiveness of the strategies.

**Discussion:**

As a result of the intervention, we anticipate that Black women within selected health centers will be more likely to receive PrEP prescriptions. During post-implementation analysis, we will identify key barriers and facilitators within the PrEP care continuum during the implementation process at the patient, provider, and system levels. This study contributes to addressing the inequity in PrEP delivery, aiming to increase initiation and persistence of PrEP for women in family planning centers. If determined effective, these implementation strategies will be further disseminated for use in similar family planning health centers and for future study in potential scale-up.

## Introduction

Black women experience one of the highest incidences of HIV in the United States yet are often not prioritized in HIV prevention efforts [1,2]. In the U.S., women account for approximately 20% of annual HIV diagnoses [3]. Twenty-three percent of all people living with HIV (PWH) are women, with heterosexual contact accounting for 86% of all transmission among women [3]. Black women represent only 13% of the national population yet account for 60% of new HIV infections among women [4]. Moreover, the rate of new HIV infections among Black women is 20 times higher than that of White women [4].

Additionally, the prevalence of HIV is high in urban areas of the Midwest, including Chicago, where the prevalence of Black women living with HIV is almost 15 times higher than White women [2]. Chicago has multiple community areas that represent a concentrated HIV epidemic, with prevalence consistently over 5% [5]. Women in these areas of Chicago remain vulnerable to HIV, even while transmission rates among women remain steady or decline throughout the U.S.

Pre-exposure prophylaxis (PrEP) has been proven to be beneficial and provide substantial protection against HIV; however, uptake among Black women has been disproportionately low. In 2015, the Centers for Disease Control and Prevention (CDC) estimated that up to 500,000 Black women in the U.S. may be eligible for PrEP based on CDC clinical guidelines, yet Black women are severely under-prescribed PrEP compared to White women [6]. Specifically, pharmacy records show that fewer than 50,000 women have initiated PrEP since 2012 when it was first approved, with less than 7,000 (14%) of those being Black women [5–7].

Our team developed and tailored implementation strategies for optimizing PrEP uptake utilizing previously identified barriers and facilitators to PrEP uptake [8–10]. Patient-level barriers included low PrEP awareness, concerns about side effects, potential impacts on fertility and pregnancy, and uncertainty about how to pay for PrEP. Provider-level barriers included lack of PrEP-specific training, need for additional clinical support for PrEP protocols, and need for additional training to increase comfort discussing PrEP with patients [8]. Health center-level implementation strategies, such as HIV prevention with family planning services, routine PrEP education for patients, standardized provider training, electronic health record (EHR) tools, PrEP navigation, and on-site clinical champions, offer a structural intervention for both patient and provider barriers. This specific set of strategies has yet to be implemented and evaluated in the context of family planning centers. Furthermore, family planning centers that provide contraception and sexually transmitted infection (STI) prevention services may be an underutilized context where women can successfully transition from awareness to initiation of PrEP. Linkage to PrEP in the context of family planning centers is critical, as the majority of women who are diagnosed with HIV in a given year are of reproductive age (i.e., 15–44 years of age) [4].

Our proposed study will implement and evaluate the impact of these adapted implementation strategies (hereafter referred to as “EUPHORIA”) within the Planned Parenthood of Illinois (PPIL) setting to improve PrEP care continuum outcomes for Black women. By identifying, adapting, and evaluating implementation strategies in PPIL health centers, we seek to address the gap in knowledge in determining effective strategies to increase uptake of PrEP among Black women.

## Materials and methods

### Aims and objectives

This study seeks to evaluate implementation strategies to address barriers and increase uptake of PrEP among women in PPIL through three specific aims. Aim 1 will adapt the PrEP implementation strategies for engaging Black women in the PrEP care continuum in eight PPIL centers. Adaptations will be informed by qualitative data collected through interviews and focus groups with PPIL administrators, staff, providers, and patients and guided by the Dynamic Adaptation Process (DAP) [11]. Aim 2 will implement the strategies that are identified in Aim 1 and tailored accordingly via a stepped-wedge cluster randomized trial (SW-CRT) across the eight PPIL center sites. Aim 3 will assess and document implementation outcomes and report on key barriers and facilitators related to the implementation of the intervention.

A mixed-methods approach including focus groups and interviews with key stakeholders will be used to evaluate implementation outcomes. This evaluation will be guided by the Reach, Effectiveness, Adoption, Implementation, and Maintenance (RE-AIM) framework and the Consolidated Framework for Implementation Research (CFIR). Findings will provide insight into implementation strategies that can support effective replication across Planned Parenthood health centers and other health centers providing family planning services nationwide to reach a greater number of Black women at risk. This study was approved by the Institutional Review Board at Rush University Medical Center (#21120602).

### Guiding theories and frameworks

The Social Ecological Theory is the guiding theory that underlies EUPHORIA. This theory highlights that an individual’s behavior and health-related decisions are embedded in an intersectional context (provider, health center, etc.) [12]. Research has consistently shown that barriers within healthcare are driven and sustained by multi-level factors and interventions that fail to consider such overlapped levels will likely fall short of achieving a comprehensive understanding of the drivers of health disparities [13–15]. The Social Ecological Theory can be leveraged to identify high-impact points within health centers and center task flow that can facilitate the successful implementation of interventions. Our proposed implementation strategies to support PrEP uptake among women address multiple levels including the individual (patient education), provider (provider training), health center system (EHR tools), and broader health system (PrEP navigation, clinical champion) (Table 1).

**Table 1:** EUPHORIA intervention package of strategies.

Strategy	Description
Routine PrEP education	PrEP education provided at each clinic visit to all Black women
Standardized provider training	3 sessions of 2-hour virtual trainings covering identifying PrEP-eligible women, working with the PrEP navigator/navigation team, and practicing difficult conversations with virtual Black women patients (avatars)
EHR tools	Incorporate the PrEP indication documentation into the EHR; provide monthly reports of PrEP continuum outcomes for clinic staff
PrEP navigation, clinical champion	Trained PrEP navigator to meet with patients to support PrEP uptake, monthly check-in with patients thereafter to support retention and adherence; follow-up appointments offered in-person or via telehealth; PrEP expert who can guide clinicians in implementing the EUPHORIA strategies

PrEP: Pre-exposure Prophylaxis; EHR: Electronic Health Record

This study will also utilize the Consolidated Framework for Implementation Research (CFIR). CFIR consists of five inter-related domains: intervention characteristics, outer setting, inner setting, characteristics of individuals involved, and the process of implementation [16]. CFIR accounts for the non-linear process of implementation and incorporates consideration of the adaptation that is required for different contexts, making it a useful framework for this study [17]. Moreover, as our study focuses on a specific, marginalized, and often overlooked population, we propose to integrate health equity domains across multiple levels of CFIR [18,19]. Health equity domains focus on multilevel factors in healthcare disparities with an emphasis on unique factors that populations experience due to social and historical marginalization, including: 1) cultural factors of recipients, 2) patient-provider interaction, and 3) societal context (including but not limited to social structural determinants of health) [20].

The Reach, Effectiveness, Adoption, Implementation, and Maintenance (RE-AIM) framework is an evaluation and reporting scheme commonly used to examine the public health impact of an intervention [21]. RE-AIM provides a useful measurement framework for capturing both clinical and implementation outcomes within the same study [22,23]. Integrating CFIR with Health Equity domains and RE-AIM within a hybrid clinical effectiveness-implementation trial will generate the evidence needed to rapidly translate our findings to standard practice.

### Setting

EUPHORIA implementation strategies will be adapted and implemented in eight PPIL health centers (Table 2). All study sites are located within Cook County, Illinois, which is a priority jurisdiction of the CDC’s Ending the HIV Epidemic (EHE) initiative [24]. These sites were chosen due to their area’s heightened HIV burden as identified by the EHE initiative and the higher volume of PrEP-eligible Black women within their patient population. All participating PPIL health centers currently have low PrEP usage (<1%) among Black women who receive care at their sites. Additionally, these sites were selected due to their varied locations within Cook County and subsequently, varied contextual factors (e.g., prevalence rates of HIV and other STIs, economic hardship index scores, etc.). By adapting and implementing the proposed strategies in multiple distinct neighborhoods within Cook County, we aim to assess the consistency and discrepancy of implementation outcomes across sites, increasing applicability of findings to a broader range of settings through exploration of site-specific contextual elements.

**Table 2:** Participating health centers and corresponding patient volume in calendar year 2023.

Planned Parenthood of Illinois Health Center	Number of unique Patients	Volume of patients who are women	Volume of patients who are Black women
Heath Center 1	3,532	2,046 (57.9%)	224 (6.3%)
Health Center 2	4,732	2,889 (61.1%)	438 (9.3%)
Health Center 3	3,462	1,128 (32.6%)	224 (6.4%)
Health Center 4	4,611	870 (30.2%)	144 (3.1%)
Health Center 5	10,214	3,970 (38.8%)	502 (4.9%)
Health Center 6	3,849	2,584 (67.1%)	518 (13.5%)
Health Center 7	4,293	1,457 (33.9%)	217 (5.1%)
Health Center 8	2,838	857 (18.8%)	144 (5.1%)

### Study design

EUPHORIA will use a SW-CRT to measure changes in the number of PrEP prescriptions initiated among Black women accessing PPIL services who are eligible for PrEP (primary clinical outcome) and the receipt of PrEP refills among those who initiate PrEP (secondary clinical outcome). In a stepped-wedge study, each health center provides observations to the control condition (standard of care) and the intervention conditions [20–22]. Each cluster switches from the control to the intervention condition at regular intervals or “steps”. The step (time point) at which a health center switches from control to intervention is randomly assigned [25]. The stepped-wedge design provides a pragmatic and robust approach to testing the effectiveness of health service delivery interventions [26]. This approach’s pragmatism arises from the rollout of the intervention over time, which enables study staff to support the implementation of a multi-pronged intervention at multiple study sites within a randomized design and increases study sites’ willingness to participate because all health centers receive the intervention during the study period. The stepped-wedge design is robust due to each cluster providing data under both the control and intervention conditions and, therefore, serving as its own control. This design improves the precision of the study and allows for intervention effectiveness to be measured across sites with different characteristics. Varied durations under the control and intervention conditions and randomly assigned transition timing enhance the statistical power in this design to differentiate intervention effects from underlying time trends.

**Fig 1:**
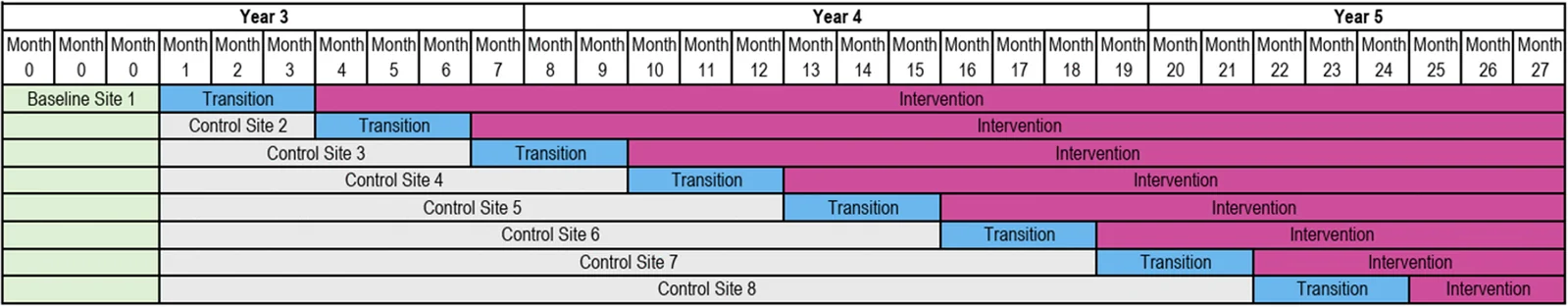
Timeline of stepped-wedge cluster randomized trial design (control, transition, and implementation periods).

In our stepped-wedge design (Fig 1), each step is three months long, and there are eight waves of intervention implementation, with one site beginning the intervention at each step [20]. The eight participating PPIL health centers will be randomized to a step, at which time they will implement the intervention. Baseline (non-intervention) data in all sites will be collected for three months prior to the first intervention step. Each health center will also undergo a three-month transition period when the health center prepares to implement the intervention package and outcome data are treated as occurring in neither the control nor intervention period.

### Study procedures

#### Pre-implementation phase.

The pre-implementation phase of the study will occur over a 12-month period prior to the intervention phase. During this phase, we will seek feedback from a variety of stakeholders to inform implementation strategy design through interviews, focus groups, and the development of a Community Advisory Board (CAB). Additionally, we will identify and engage with clinical champions at each PPIL health center to collaborate on recruitment of focus group and individual interview participants and design of local implementation strategies. The health center clinical champion will participate in regular meetings with the study team and help identify eligible staff and patients for the interviews.

For staff key informant interviews, we will recruit diverse staff members from the eight participating health centers with varying backgrounds, roles, and perspectives such as administrators, medical assistants, and providers. The interviews will focus on staff’s perspectives on barriers and facilitators to the implementation of the proposed strategies into clinical practice and any adaptation or resource considerations. Staff and providers will be recruited using informational flyers and in-person invitations by research staff and with the assistance of the health center’s clinical champion, PPIL’s Director of Research, and PPIL research assistants. Staff interested in participating will complete an eligibility screener via the informational flyer or otherwise contact the study team. Staff will be eligible to participate if they are (1) 18 years of age or older, (2) currently employed at a participating PPIL health center, (3) able to speak and understand English, and (4) willing and able to give consent. Interviews will be conducted by the study team either in-person at a health center or virtually via Zoom with audio recording only and no collection of personally identifiable information. Interviews will last approximately 30–60 minutes, and participants will receive a $50 incentive via physical or electronic gift card.

In addition to staff interviews, we will conduct interviews and focus groups with Black women seeking care at participating PPIL health centers. These interviews will be conducted to identify women’s preferences and needs regarding PrEP and to obtain specific feedback on the proposed implementation strategies and integration process. Active recruitment and enrollment of eligible patients will take place at each PPIL health center; staff from PPIL and members of the study team will recruit patients in the waiting area or lobby of each health center to inform them of the study. Passive recruitment will also occur via flyer distribution and postings, which will contain a QR code link to the eligibility screening survey and study team contact information. Patients will have the opportunity to complete the eligibility screening survey in person or remotely via the link. Patients will be eligible to participate if they are (1) 12 years of age or older; (2) Black/African American women; (3) a current patient of a participating PPIL health center, (4) HIV-negative or have unknown HIV status based on self-report, (5) able to speak and understand English, and (6) willing and able to give consent. Upon completion of the eligibility screening survey, participants will be presented with the choice to participate in a focus group or a key informant interview. Interviews will be conducted by the study team either in-person at a health center or virtually via Zoom with audio recording only and no collection of personally identifiable information. Interviews will last approximately 60 minutes, and participants will receive a $50 incentive via physical or electronic gift card.

To reflect the communities that we will be working with and to center the needs and perspectives of Black women, we will create a CAB during the pre-implementation phase, with representation from Black women of reproductive age and varied PrEP experience who seek care at PPIL. The CAB will consist of a facilitator who is a leader in PrEP and HIV advocacy in the Chicago area, as well as 6–8 community members who live in Cook County, Illinois and seek services at PPIL. The CAB will engage in a planning and advisory role throughout the study duration by guiding the study approach and ensuring that the implementation strategies are tailored appropriately for Black women. The CAB members will meet quarterly throughout the study duration. Each member will be compensated $100 per meeting.

#### Implementation and post-implementation phases.

All outcome data to be collected are part of existing routine EHR data collection at PPIL. PPIL Information Technology (IT) will create custom reports in the PPIL data warehouse to be built utilizing the existing data fields and a table of desired variables (Table 3).

**Table 3:** Requested electronic health record variables within limited data set.

Data domain	Brief description
Denominator/overall PrEP eligibility	Number of patients who are eligible to learn more about and receive PrEP according to study criteria: all patients 12 years or older, who are not living with HIV, and who have had a relevant medical encounter (e.g., family planning, sexual health care, primary care, etc.) during which PrEP could be prescribed *PrEP ineligibility: Patient is less than 12 years old or is medically unable to use PrEP (e.g., living with HIV) or attends a medical encounter during which PrEP is unlikely to be discussed or prescribed.
PrEP eligibility characteristics	STI screening history, risk factors, prior PrEP visits, and HPI for PrEP, visit date, visit type, location of care
Primary and secondary outcome measures	
PrEP initiation (i.e., initial prescription) – primary outcome	Proportion of Black women at each site who receive an initial prescription for PrEP (i.e., have not received a prescription for PrEP in the prior six months)
PrEP persistence (i.e., subsequent/refill prescription) – secondary outcome	Proportion of Black women at each site who receive a subsequent prescription of PrEP after initiating PrEP (i.e., refill initial prescription within six months)
Process measures	
HIV risk assessment	Proportion of eligible Black women who undergo HIV risk assessment at each site
PrEP education	Proportion of eligible Black women who receive PrEP education at each site
PrEP navigation	Proportion of eligible Black women who are referred to PrEP navigator at each site
Sociodemographic data/covariates	Gender identity, sex assigned at birth, sexual orientation, race, ethnicity, date of birth, gravidity, parity, annual income, preferred language, home zip code, marital status, disability status, HIV status, STI testing and diagnosis history within past year, location of care, prescription name(s) and duration(s)

PrEP: Pre-exposure Prophylaxis; STI: Sexually Transmitted Infection; HPI: History of Present Illness

Reports will be built to monitor (1) EHR alert consistency, (2) if PrEP is offered, (3) if PrEP is accepted or declined, and (4) among those who accept, if patient navigation is utilized and if PrEP is prescribed. These data will be collected at the health center level and during two phases: 1) the control, or baseline phase and 2) the intervention phase. The control and intervention phases are of variable length depending on step assignment (Fig 1). Implementation outcomes of each health center will be assessed at the midpoint and endpoint of the intervention period.

The initiation of PrEP prescriptions for eligible Black women (primary clinical outcome) will be measured using de-identified patient-level EHR data. Eligibility for inclusion in the outcome measures among patients will be determined based on study inclusion criteria such as patient’s age, biological sex and gender identity, and race. The primary outcome will be the proportion of eligible Black women at each health center who receive a prescription for PrEP during that month. The secondary outcome will be PrEP refills within six months, i.e., receipt of a PrEP prescription within six months of the index visit among those in the eligible population with a prior PrEP prescription. Finally, the total number of PrEP prescriptions among all patients in the control (baseline) and intervention periods will be analyzed to determine the impact of the implementation strategies on the entire PPIL patient population.

At approximately the midpoint of each site’s intervention period, key informant interviews including focus groups and interviews will be conducted with PPIL staff, clinicians, and women to assess implementation outcomes such as acceptability, feasibility, and fidelity over time. These interviews will elicit information related to the barriers and outcomes encountered during implementation. Procedures for recruitment and enrollment for these participants will mirror those of pre-implementation qualitative data collection completed as part of Aim 1. Data will be coded according to the RE-AIM constructs, with emerging themes identified and categorized by implementation outcome: acceptability, feasibility, and fidelity (Table 4). The usage of mixed methods in this study will be used to elaborate on quantitative RE-AIM findings with themes from qualitative data. Participant recruitment and data collection are expected to be completed by June and December 2028, respectively.

**Table 4:** RE-AIM outcomes.

RE-AIM dimension	Data source	Outcome
Reach	EHR	Percent of PrEP eligible women identified
Effectiveness	EHR	Percent of eligible women receiving PrEP prescription
Adoption	EHR	Percent of eligible women offered PrEP
Implementation	EHR Key informant interviews	Acceptability and feasibility of intervention as reported by staff, clinicians, and patients
Maintenance	EHR	Reach and implementation over time(Timeline: Months 13–60)

RE-AIM: Reach, Effectiveness, Adoption, Implementation, and Maintenance; EHR: Electronic Health Record; PrEP: Pre-exposure Prophylaxis

#### Sample size.

This study will be powered to determine whether there is a statistically significant difference in PrEP prescriptions among at-risk Black women defined as those with a positive STI test accessing family planning services between the intervention and control periods. We choose to use presenting to a family planning clinic and a positive STI test (gonorrhea, chlamydia, or syphilis) as eligibility to ensure patients at high risk for HIV acquisition are offered PrEP within the PPIL context, keeping in line with the CDC’s guidance to offer PrEP to patients at substantial risk of HIV acquisition. A power calculation was conducted based on a mixed effects effectiveness model with a random intercept for clinic and intervention and time as fixed effects. Assuming a baseline PrEP uptake of 1% and a conservative intraclass correlation coefficient (ICC) of 25%, a trial with 10 PrEP-eligible Black women per month (30 women per step) at each of the 8 clinics (8 steps) would allow us to detect an increase to 5% PrEP uptake (corresponding to an odds ratio of 5.21 for the intervention effect) in eligible Black women following the introduction of the intervention with >90% power at a two-sided alpha of 0.05. Our sample size provides almost 100% power for detecting a larger increase to 15% PrEP uptake (corresponding to an odds ratio of 17.47 for the intervention effect). Our sample size accounts for both the clustered nature of the design and the potential confounding effect of time. We used R software for power analysis.

#### Informed consent.

A waiver of parental permission was provided for Aim 1 and Aim 3 of this study. Under the statutory authority of Illinois State Law (410 ILCS 210), minors in this specific age category (12–17, inclusive) may consent on their own authority to participate in this research study.

No individual patient consent or recruitment is required for activities related to Aim 2 of this system-level, clinic-level health service delivery intervention, and therefore outcomes may be obtained through routinely collected de-identified data from the PPIL EHR.

### Analysis

#### Qualitative data.

Data collected from focus groups and key stakeholder interviews with patients (see S1 File) and staff (see S2 File) during the pre-implementation, implementation, and post-implementation phases will be thematically coded according to CFIR and RE-AIM constructs using Dedoose software. Emerging themes will be identified and categorized by construct and/or implementation outcome and used to create a codebook with unique definitions and examples for each code. The data will be coded by at least two independent coders then discussed to resolve coding discrepancies and to maintain high inter-rater reliability (80% agreement). During pre-implementation, emerging themes will be identified and categorized as important considerations for implementation planning, highlighting key content and contextual modifications; these adaptations may include refining to make intervention strategies more appropriate or applicable, adding/removing/substituting intervention components, or adjusting pacing. For instance, adaptations may include adjusting the frequency, duration, and content of the provider training sessions. Similarly, modifications to EHR optimization will aim to enhance existing processes for PrEP screening, prescribing, and navigation. Patients will also be asked about their experiences and perceptions of existing PrEP educational materials during interviews to guide updates that reflect patient preferences and improve relevance for various populations.

During implementation and post-implementation, emerging themes will provide feedback on the strengths and weaknesses of the intervention based on the RE-AIM framework to improve future implementations and assess implementation processes. Additionally, the Framework for Reporting Adaptations and Modifications (FRAME) will be used to facilitate comprehensive documentation of adaptations made to illustrate significant mechanisms and processes in implementation [23].

#### Quantitative data.

We will compare the distribution of patient characteristics between the unexposed observations from the control period and the exposed observations during the intervention period overall and within each center to identify potential confounders. Then, we will plot rates of initial (primary clinical outcome) and refilled PrEP prescriptions (secondary clinical outcome) over each time period, comparing how rates differ from the control period to the intervention period across all centers and within each center, to examine the effects of the intervention site and time on the outcomes. To examine the treatment effect, a generalized linear mixed-effects model will be used, with a random intercept for each PPIL site and fixed effects for the intervention and for each time interval [27], where the fixed effect for each time interval is included to account for secular trends. Any identified potential confounders will also be included as fixed effects in the model. An intention-to-treat analysis will be used to ensure a conservative estimate of the intervention effect in the case that a site deviates from the assigned intervention timeline or has low intervention fidelity. Additionally, any identified potential mediators will be investigated. We will carefully monitor the timing and measure of the impact of the availability of new PrEP modalities (i.e., long acting injectables) on intervention effects. Their potential influence on intervention effects will be addressed by including indicator variables for the time periods in which these PrEP modalities become available. We will adopt complete case analysis to deal with missing covariates in the primary analysis. To assess the robustness of our findings, we will perform sensitivity analyses using multiple imputation models that account for clustering by site, study period, and intervention status [28]. Additional exploratory sensitivity analyses will include missing-indicator approaches for covariates, acknowledging their limitations and the potential for bias. We anticipate all results to be available by December 2029.

## Discussion

As a result of the intervention, and per our sample size calculation, we anticipate that Black women within selected PPIL health centers will be more likely to receive PrEP prescriptions following implementation, contributing to an anticipated increase in PrEP uptake of up to 15 percent. This magnitude of impact would be statistically significant and clinically meaningful, supporting scale-up through PPIL and other family planning centers. The present study design is powered to detect small increases during the study period and will support future exploratory analyses to examine trends over time. During post-intervention analysis, we anticipate identification of important barriers and facilitators as a continuum during the implementation process at the patient, clinician, health center, and system levels.

As the duration of the intervention period will differ by health center due to the SW-CRT design, implementation outcomes will be assessed at various stages at the different study health centers. This variance will be a strength in analysis in that a wider variety of implementation experiences will be available to us; however, the health centers with the shortest intervention periods may provide somewhat limited evidence for measures such as implementation and maintenance. Throughout the study, we will monitor and evaluate procedure adaptations and modification as well as implementation processes at each health center; this support will include facilitating provider training, providing technical and logistical assistance such as EHR support, and serving as a resource for PrEP navigators and clinical champions. Additionally, we will measure the acceptability and fidelity of the intervention package to document strengths and weaknesses while determining the progress on achieving the primary and secondary clinical outcomes through regular analysis of EHR data.

Although the SW-CRT design is well-suited for this study due to its pragmatism and robustness, we also anticipate some design-related challenges will arise throughout the implementation process. In SW-CRTs, challenges such as structural limitations to adopt strategies may occur within the health centers. Additionally, changes to the planned timeline for the trial (lengthened or shortened duration), delayed or early implementation in clusters, or other logistical and technical issues (e.g., staff turnover, potential contamination across sites, health center resistance to intervention, loss of resources, etc.) can be significant challenges in SW-CRTs [29]. The randomization of health centers to various steps may also cause tension or frustration with partners due to health centers receiving the intervention at different times. To mitigate and address these potential challenges, we have incorporated a three-month transition period immediately preceding implementation of the strategies at each health center to ensure staff are prepared and have the necessary resources for implementation.

Additionally, the transition period serves as an opportunity to anticipate and prepare for any potential health center-specific challenges and to streamline implementation of PrEP uptake strategies, enabling a shift from standardized practices to increased comfort with utilizing the new systems and talking about PrEP among PPIL’s leaders, clinicians, staff, and patients. The one-year pre-implementation period will be utilized to build rapport and trust from health center staff to improve buy-in and develop relationships that will support implementation. For example, the pre-implementation processes will include the identification of at least one clinical champion to serve as a liaison between the health center staff and study team; the developed relationship with the clinical champion in particular will be leveraged to identify and address staff concerns or need for clarification or re-education and to gain on-the-ground knowledge to more effectively support centers throughout the duration of the study. Finally, any deviations and modifications to the study design and procedures will be documented in a tracking framework developed by the study team based on FRAME to help measure fidelity to the intervention [26].

By investigating effective implementation strategies for prevention of HIV infection among Black women, specifically in the EHE priority jurisdiction of Cook County, IL, the findings of this study will support women at increased need for HIV prevention using evidence-informed interventions. Understanding identified barriers and facilitators to the implementation and effectiveness of EUPHORIA could help improve not only PrEP delivery at local PPIL health centers but also understanding of similar interventions within other family planning centers and the larger Planned Parenthood Federation of America. Upon successful implementation, these data will inform the next steps, including to extend lessons learned within similar health centers to broaden reach and to scale up the intervention package. This study may also serve as a foundation for future studies of PrEP initiation and persistence in broader, more diverse settings. The findings of this study will be disseminated to participating health centers via written reports and presentations and to the larger community in the form of conference abstracts and publications in peer-reviewed journals in an effort to reduce the gap in PrEP delivery and uptake, share effective implementation strategies for use among a key population, address disparities in access to HIV prevention care, and contribute to the overall elimination of HIV transmission, especially among Black women.

### Trial status

The study is registered under NCT06335121. The SW-CRT will take place over the course of 27 months, with implementation strategies being rolled out at one site every 3 months. The research team will modify procedures as needed to ensure successful implementation at each health center throughout the duration of the trial.

## Supporting information

S1 File Qualitative interview guide for patients is included as Supporting Information file S1. (DOCX)

S2 File Qualitative interview guide for providers/staff is included as Supporting Information file S2. (DOCX)
