# Supplementary material for: Enhancing uptake of PrEP for her via organizational research and individual actions (EUPHORIA): Protocol for a stepped-wedge cluster randomized trial
Source: PLoS One. 2026 Aug 27;21(8):e0355623. doi: 10.1371/journal.pone.0355623 (PMC13521356; doi:10.1371/journal.pone.0355623)
Supplement: Supplementary file 1 [file pone.0355623.s001.docx]

**PROTOCOL TITLE:** Enhancing Uptake of PrEP for Her via Organizational Research and Individual Actions (EUPHORIA)

**INTERVIEW GUIDE – PATIENTS**

**OBJECTIVES:**

- **Understand patient’s knowledge and experience of PrEP**
- **Understand patients’ thoughts on PrEP education, being offered, PrEP, and PrEP navigation services**
- **Understand and explore contextual factors influencing clinic environment and decision making about PrEP**

**FORMAT:**

Qualitative Interview (60 minutes)

*[Interviews will only occur after each patient has consented to participate]*

**INTRODUCTION:**

*Introduction of interview facilitator (designated research staff)*

Today we are going to be talking about your knowledge, experiences, and opinions about pre-exposure prophylaxis (PrEP). We will share some patient education materials for your review and feedback. First, I am going to ask you some questions about your experiences in community health clinic (specific site name) and then transition into talking about your knowledge about a specific HIV prevention strategy called PrEP.

- Please leave your cameras off and do not use your real name.
- Audio recording is for research purposes and for internal use only. When we are finished, the audio files will be uploaded to a secure transcription service so someone can type out every word that was said. Then, we will destroy all of the recordings.
- I will be asking lots of questions. It is important to remember that there are no right or wrong answers, but I might ask you to elaborate and explain your answers. For example, I may ask a lot of “Why?” questions for explanations to clarify your answer and make sure I understand your thoughts. There is no judgment in our conversation.
- Everything you say during this discussion is considered confidential. I want you to feel as comfortable as possible while telling me your thoughts and opinions.

**INTERVIEW CONTENT:**

- How would you describe your use of the community health clinic (*specific site name*) within the past year?
  - *Probe:* Uses family planning clinic as primary care site, visits family planning clinic often, only uses family planning clinic for certain health problems/testing, etc.
- How would you feel if you were asked about HIV and sexual health during a clinic visit?
  - *Probe:* Would rather focus on presenting complaint, want to talk but only if providers initiate the conversation, don’t think it is necessary, recent negative HIV/STI test, feel embarrassed or afraid, don’t want to bother the provider, concerned about confidentiality, depends on who is asking, etc.
- Have you ever heard of PrEP? If so, what have you heard about it?

***(Give explanation of PrEP)***

- *Probe*: indications, efficacy, side effects, etc.
- *Probe:* What do you think about PrEP - would you use it? Why or why not?
- *Probe:* Do you know anyone who takes PrEP?
- Has any clinic staff member ever talked to you about PrEP?
  - *Probe:* Who do you think is the best person to discuss PrEP with patients? (e.g., primary care providers, OB/GYN providers, ED providers, PrEP navigators/staff, all healthcare providers, etc.)
  - *Probe:* Describe the clinic environment when it comes to PrEP - specifically, are there any posters, handouts, or information for patients about PrEP?
  - *Probe:* What are your recommendations for the best way to inform Black women about the availability of PrEP at (*specific site name*)?
- If a clinic doctor or other staff told you that PrEP might be a good option for HIV prevention, how would that make you feel?
  - *Probe:* What concerns or worries you about being identified as at risk for HIV or vulnerable to HIV while in clinic?
  - *Probe:* What would make this conversation easier to have? What advice would you give doctors? What advice would you give other women?
- We are interested in talking about different medical settings and types of appointments in which PrEP could be offered.
  - What do you think about offering PrEP in Pediatric visits? For example, offering PrEP to adolescents or linking parents bringing adolescents to appointments to PrEP?
  - *Probe:* Prenatal visits? Post natal visits?
    - Linkage for mothers during well child visits
  - *Probe:* Ob/gyn visits?
- In the past few years there has been a rise of online access to medication and mental health services, such as “BetterHelp.com” and “hers”. These online services link patients to a virtual doctor’s visit that is often covered by insurance or is a low out-of-pocket fee and then link the patient to medication. If a service like this was available for PrEP, do you think women would use it?
  - How could we let women know the service was available?
  - What are good things about this approach? What are some bad things?

**PART 3. Reactions to PrEP Patient Education and PrEP Navigation**

Now I want to go over some of the content of existing PrEP education. I’m interested in your feedback on how we can adapt the content to best serve Black women. Let’s start with an outline of the content.

**Topics covered in Patient education materials**

- What is PrEP?
- Who can take PrEP (Who is eligible for PrEP)?
- How do you get PrEP?
- How much does PrEP cost?
- Payment Resources
- For the Insured
- Copayment Assistance for the Insured
- For the Uninsured and the Underinsured
- What to expect taking PrEP.
- What you will need to bring to your first appointment
- First appointment
- Follow-up appointments
- Adherence- Taking PrEP Everyday

Now that I’ve shared the topics, are there any questions you have about PrEP that you think should be covered in patient education materials? (generate FAQ)

**Educational Materials for Patient**

Now I am going to share my screen and show you some images of existing flyers and pamphlets. We won’t go into all the details of the text, but I want to go through the images and overall content.

**[Show POWER-Up Flyer]**

- What do you think about this flyer? Does this flyer resonate with the population we are trying to reach (Black Cis-women)
- How do you feel about the branding and messaging of this flyer?

- In your opinion, what are the benefits or the good things about this material?
  - *Probe:* format, message
- Is there anything that concerns or worries you about this material?
  - *Probe:* format, message
- How do you think other people in your community would feel about this material?
- Do you have any suggestions for additional material or strategies to support women in understanding PrEP?
- What other formats or modes can we use to make sure women are getting information and education on PrEP?
  - *Probe:* websites, social media, etc.

**Part 4. PrEP Support**

Now I want to talk about support for starting PrEP.

- If you were to decide to take PrEP, what help or support do you think you would need?
  - *Probe*: Figuring out how to pay for PrEP? Managing appointments? Questions about the medication? Managing adherence?

PrEP navigation is a service where the clinic would link you to a staff member who could help you navigate getting on PrEP, paying for it, refilling your prescription, and managing taking PrEP.

- What would you think if a clinic doctor or another staff member referred you to a PrEP navigator (other staff to help assist with HIV prevention)?
- In your opinion, what are the benefits or the good things about PrEP navigation?
  - *Probe*: What would make it easier to talk to a PrEP navigator? Would their race, gender, age, or other identity make a difference? What would make it hard to talk to a PrEP navigator? What would you think if the PrEP navigator was a peer?
- Is there anything that concerns or worries you about PrEP navigation or receiving support for PrEP?
- Is there anything else you’d like to share about receiving PrEP information or support for PrEP?

**Part 5. PrEP modalities**

In addition to daily oral pills, there are other modes of PrEP available. Long-acting injectable cabotegravir is administered by a nurse via injection into the buttock muscle every two months. This injection is highly effective in reducing the risk of HIV infection, with efficacy approaching 100%. After the initial injection, the drug can remain in the body for up to one year. While the first injection may be painful or with discomfort, subsequent injections are generally better tolerated. The treatment is safe during pregnancy. There are no major side effects but CAB-LA can lead to weight gain (~1.5kg the first year).

- What do you think about this every 2-month injection?
- What questions/concerns would you have about this method?
- Compared to daily oral PrEP, which mode do you prefer?

Long-acting injectable lenacapavir is administered by a nurse under the skin (like a vaccine) of the belly every 6 months. This injection is highly effective in reducing the risk of HIV infection, with efficacy approaching 100%. Injections can result in pain, discomfort and let persistent nodules under the skin for 60% of women. There are no other major side effects. After the initial injection, the drug can remain in the body for up to one year. We don’t know if the treatment is safe during pregnancy.

- What do you think about this every 6-month subcutaneous injection?
- What questions/concerns would you have about this method?

In the coming years, a significant advancement in women's health is on the horizon with the potential development of a dual prevention pill that combines PrEP for HIV prevention with contraception. It will be a daily pill offering a convenient and effective solution for women seeking to protect themselves from both HIV infection and unintended pregnancy.

- What do you think about this dual prevention pill?
- What questions/concerns would you have about this dual prevention pill?
- When you think of all these new developments in the field of PrEP, how would you rank them from most to least useful? Which one would you try and why?

**Part 6. Thank You**

Thank you for your time and for sharing your thoughts, opinions, and experiences with us. You will receive your e-gift card as soon as possible.
