# Supplementary material for: Enhancing uptake of PrEP for her via organizational research and individual actions (EUPHORIA): Protocol for a stepped-wedge cluster randomized trial
Source: PLoS One. 2026 Aug 27;21(8):e0355623. doi: 10.1371/journal.pone.0355623 (PMC13521356; doi:10.1371/journal.pone.0355623)
Supplement: Supplementary file 2 [file pone.0355623.s002.docx]

**PROTOCOL TITLE:** Enhancing Uptake of PrEP for Her via Organizational Research and Individual Actions (EUPHORIA)

**INTERVIEW GUIDE – STAFF**

**OBJECTIVES:**

- Understand previous training/education experiences regarding pre-exposure prophylaxis (PrEP) and current trainings/education offered at Planned Parenthood
- Understand existing PrEP patient education (informational materials and process of educating clients, any differences for educating women or Black cisgender women specifically)
- Preview existing intervention material and discuss how contextual factors may inform adaptation, with specific considerations for Black cisgender women

**FORMAT:**

Qualitative Interview (60 minutes)

*[INTERVIEWS WILL ONLY OCCUR AFTER EACH PARTICIPANT HAS CONSENTED]*

**INTRODUCTION:**

*Introduction of interview facilitator (designated research staff)*

Today we are going to introduce the EUPHORIA implementation strategies and talk about how we might modify the strategies to better fit your specific Planned Parenthood site. We would like to learn about the PrEP workflow as well as any interventions that your site may be implementing to improve PrEP uptake in general and specifically among Black cisgender women (hereafter referred to as “women”). We will also discuss any PrEP training you have participated in previously. Finally, we will preview some EMR flags, reports, and ideas for better using the EMR to support PrEP uptake. The purpose of the interview is to learn as much as we can about what currently exists at your site and how we can adapt EUPHORIA to the needs of Black women who receive care at Planned Parenthood. Before we begin, we will go over a few general rules.

- Audio recording is for research purposes and for internal use only. When we are finished, the audio files will be uploaded to a secure transcription service so someone can type out every word that was said. Then, we will destroy all of the recordings.
- I will be asking a lot of questions. It is important to remember that there are no right or wrong answers, but I might ask you to elaborate and explain your answers. For example, I may ask a lot of “Why?” questions for explanations to clarify your answer and make sure I understand your thoughts. There is no judgment in our conversation.
- Everything you say during this discussion is considered confidential. I want you to feel as comfortable as possible while telling me your thoughts and opinions.

**INTERVIEW CONTENT:**

Okay, let’s get started. The first thing we are going to do is introduce the implementation strategies we proposed as part of this project, which are: provider training on PrEP, patient education, EMR optimization, PrEP navigation, and clinical champions. By implementing these strategies, we hope to increase PrEP uptake to 15% among eligible Black women following the introduction of the study.

**IMPLEMENTATION STRATEGY: PROVIDER TRAINING**

Now that you’ve heard about our project, we want to know more about how familiar you are with PrEP and what kind of training, if any, you’ve had on the topic.

- How familiar are you with PrEP? **(*Give explanation of PrEP if necessary*)**
  - FOR PRESCRIBERS ONLY - *Probe:* Have you ever prescribed PrEP at Planned Parenthood?
    - FOR PRESCRIBERS ONLY - *Probe:* **If yes**, have you ever prescribed PrEP to a Black woman?
    - FOR PRESCRIBERS ONLY
      - *Probe:* **If no**, why not? (unfamiliar, uncomfortable, unsure about side effects/follow-up care, etc.)
      - *Probe:* What factors may prevent you from prescribing PrEP to someone?
- Have you completed any PrEP-related training?
  - *Probe:* **If yes**, can you describe the training and content? What did you learn about PrEP? How effective was the training?
  - *Probe:* **If yes**, how? - self-guided online training, in-person training, webinar, written material, other (any format, conference lecture with emailed resources)
  - *Probe:* **If yes***,* was there anything in the training that was specifically tailored to women overall or Black women specifically?
  - *Probe:* **If no**, why? (unnecessary, uninterested, not relevant to role, no time, not offered, etc.)

For this project, we proposed having three, 1-hour interactive virtual training sessions using case-based scenarios. We are exploring the option of offering CME or MOC credit. What are your thoughts on this format? Any suggested revisions?

- What are your thoughts on the training incorporating interactive role plays, to support practicing having conversations with Black women about PrEP?
  - In thinking about your specific family planning center environment, how should the training be offered? (modality, in person vs. remote)
  - *Probe:* who should be trained (e.g., all clinical staff vs. just prescribers); how often should training be offered?
  - *Probe:* What type of ongoing feedback or support do you recommend we provide after the training?

**EXISTING PROVIDER TRAINING OVERVIEW:**

Now I want to go over some of the content of the existing provider training. I’m interested in your feedback on how we can adapt the content to best serve clinicians and other staff at your site. Let’s start with the learning objectives of the content, and a brief outline.

**Learning Objectives**

- Understand risk factors of HIV transmission
- Summarize the guideline recommendations for PrEP
- Increase knowledge about providing PrEP to adolescents
- Apply recommendations for PrEP to case examples

**These are the topics that are covered in the current PrEP training at PPIL:**

- Overview of PrEP
- How to obtain a relevant history
- How to identify a good candidate for PrEP
- Guidelines for prescribing PrEP and counseling PrEP at subsequent follow-up visits
- Considerations about PrEP specifically for cisgender women

Are there any other topics that you think would be important to cover in a PrEP training for women?

- *Probes:* What type(s) of PrEP are available to prescribe at PPIL? Are you comfortable with the guidelines for prescribing both oral and injectable PrEP? Insurance/cost issues?

**Case Scenarios and Discussion**

Next, I’d like to describe some case scenarios and hear your feedback on them. Specifically, your thoughts on whether the scenarios are relevant based on your experience at PPIL or if they should be modified. And whether there are scenarios you would like to review that have not been presented.

- Case #1: A woman who is involved in sex work reports having STI symptoms, but is HIV-negative. You treat her symptoms. What would be your recommendations for her?
- Case #2: A woman has a partner living with HIV who often disengages from care. She mentions that she plans to get pregnant in the next 3 months. What would you recommend?
- Case #3: You are meeting with a new female patient who wants a prescription for birth control. Would you discuss any other sexual health topics with her?
- Case #4: An adolescent patient discloses that she has begun having sex with inconsistent condom use. Would you recommend PrEP?

Is there anything else you’d like to share on the topic of provider training?

**IMPLEMENTATION STRATEGY: PATIENT EDUCATION**

Now I am going to ask you some questions about providing routine PrEP education to clients at your family planning center. We will also discuss some patient education materials that exist, including opportunities for improvements.

- How do you discuss HIV prevention options with patients at Planned Parenthood? When does that come up?
  - *Probe:* Who do you decide to discuss PrEP with?
  - *Probe:* Do you reference the patient STI Risk Screening during their check-in? How so?
- Can you describe the information or education about PrEP that is currently available to clients at your center?
- What delivery methods are used by your center to educate clients on HIV and PrEP? (ex. Social media, visuals, flyers, videos, classes, counseling)
- *Probe:* Do you have any opinions on which method is most well-received by patients?
- Who educates clients about PrEP? (e.g., nurses, prescribers, Mas, etc.)
- Do you feel comfortable educating clients about PrEP? Why or why not?
- *Probe:* Are there any differences in your comfort level talking about PrEP with Black women specifically?
- *Probe:* Are there any differences in your comfort level talking about PrEP with adolescents specifically?

**Topics covered in print patient education materials**

The current topics are covered in print-based materials we have for healthcare centers.

- What is PrEP?
- Who can take PrEP (Who is eligible for PrEP)?
- How do you get PrEP?
- How much does PrEP cost?
- Payment Resources
- For the Insured
- Copayment Assistance for the Insured
- For the Uninsured and the Underinsured
  - Is there a state program that covers PrEP for uninsured or underinsured clients?
- What to expect taking PrEP
- What you will need to bring to your first appointment
- First appointment
- Follow-up appointments
- Adherence
- We will also be interviewing clients, but from your initial review, are there any other topics you think women or clients in general would like information on?
- Are there any topics that you think should be removed?
- How can we best tailor the information for Black women?

**IMPLEMENTATION STRATEGY: EMR OPTIMIZATION**

We are interested in learning about any current tools within the EMR that help to identify patients who are good candidates for PrEP and to prescribe PrEP, order the appropriate labs, and keep track of ongoing conversations about PrEP or PrEP follow-up appointments. We also want to get your feedback on what could be added to the EMR to make these processes easier.

- Does your EMR currently allow you to screen for PrEP eligibility?
  - *Probe:* **If yes,** how?
  - *Probe:* Do you have any suggestions for how to improve your EMR screening to capture PrEP eligible patients? (example: BPA alert that indicates someone had a recent STI and should be offered PrEP)
- Does your EMR have smart phrases or dot phrases to provide patient PrEP education?
  - Probe: **If yes,** what do they include?
  - Probe: **If yes,** are they personal dot phrases that you created, or are they available across the PPIL system?
    - If they’re not already implemented across the system, do you have any suggestions for what should be?
  - Have you provided PrEP education in your patients after visit summaries?
    - Probe: **If no,** do you think that would be helpful?
- Can your EMR currently automate a patient’s STI follow up visit with PrEP education?
  - *Probe:* What potential changes would you recommend?
- Can you easily review if you have previously discussed PrEP with a patient? If not, would you be interested in having that as an option?
  - *Probe:* Would it helpful to have a structured field with the options of PrEP discussed, PrEP declined by patient, PrEP initiated, PrEP not discussed, etc.?
- Once a patient starts PrEP, are there specific templates or workflows for follow-up appointments? If not, would you be interested in having that as an option?
  - *Probe:* For example, having a lab order set that would include all of the baseline and monitoring lab tests required for someone on PrEP?
- Are there any other tools or modifications within the EMR that you think would be helpful for capturing all steps of the PrEP continuum (identification of someone with indications for PrEP to persistence)?

**IMPLEMENTATION STRATEGY: CLINICAL CHAMPIONS**

Next, we are going to discuss clinical champions. A clinical champion is someone who is designated to support various projects within the clinic and understands what makes your clinic unique. Specifically, we are considering how having a clinical champion within Planned Parenthood can support staff and providers implement strategies that improve PrEP uptake.

- Have you worked with a clinical champion before?
  - *Probe:* **If yes,** how was your experience?
- Do you think it could be helpful working with a clinical champion who you can reach out to with questions regarding the EUPHORIA implementation strategies?
- How do you think a clinical champion can support providers and staff engaging with patients who are either interested in or currently on PrEP? Can you think of any ways that would make it easier or more useful to engage with a clinical champion?
  - *Probe:* For example, do you think it would be helpful to meet with the clinical champion on a regular basis?
  - *Probe:* Would it be appropriate/acceptable for them to attend site or provider meetings to share updates on the number of women who were prescribed PrEP and reminders of what is available to help providers prescribe PrEP to their patients?

**IMPLEMENTATION STRATEGY: PREP NAVIGATION**

Our final topic is related to the role of a PrEP navigator in Planned Parenthood Health Centers.

- What do you understand the role of a PrEP navigator to be?
- Have you worked with a PrEP navigator?
  - *Probe:* **If yes,** has it been helpful? How so?
  - *Probe:* Do you have any suggestions on how to make it more helpful?
  - *Probe:* **If you have not worked with a PrEP navigator,** do you think it could be helpful to you? How so?
- Do you think there is opportunity for a clinical champion and PrEP navigator to work collaboratively in engaging patients in the PrEP care continuum?
  - *Probe:* **If yes,** how so?
- What do you think a PrEP navigator should be responsible for vs. a physician or other provider in regard to the process of getting a patient to start and persist on PrEP?

**THANK YOU:**

Thank you for your time and for sharing your thoughts, opinions, and experiences with us. You will receive your e-gift card as soon as possible. We will contact you to make sure you received your incentive.
